# Optimal cutoff of the waist-to-hip ratio for detecting cardiovascular risk factors among Han adults in Xinjiang

**DOI:** 10.1186/1471-2261-14-93

**Published:** 2014-07-29

**Authors:** Shuang-Shuang Li, Shuo Pan, Yi-Tong Ma, Yi-Ning Yang, Xiang Ma, Xiao-Mei Li, Zhen-Yan Fu, Xiang Xie, Fen Liu, You Chen, Bang-Dang Chen, Zi-Xiang Yu, Chun-Hui He, Ying-Ying Zheng, Nuremanguli Abudukeremu, Jialin Abuzhalihan, Yong-Tao Wang

**Affiliations:** 1 Department of Cardiology, First Affiliated Hospital of Xinjiang Medical University, Urumqi 830054, China; 2 Xinjiang Key Laboratory of Cardiovascular Disease Research, Urumqi, China

**Keywords:** Cutoff, Waist-to-hip ratio, Cardiovascular risk factors, Han adults, Xinjiang

## Abstract

**Background:**

The optimal cutoff of the waist-to-hip ratio (WHR) among Han adults in Xinjiang, which is located in the center of Asia, is unknown. We aimed to examine the relationship between different WHRs and cardiovascular risk factors among Han adults in Xinjiang, and determine the optimal cutoff of the WHR.

**Methods:**

The Cardiovascular Risk Survey was conducted from October 2007 to March 2010. A total of 14618 representative participants were selected using a four-stage stratified sampling method. A total of 5757 Han participants were included in the study. The present statistical analysis was restricted to the 5595 Han subjects who had complete anthropometric data. The sensitivity, specificity, and distance on the receiver operating characteristic (ROC) curve in each WHR level were calculated. The shortest distance in the ROC curves was used to determine the optimal cutoff of the WHR for detecting cardiovascular risk factors.

**Results:**

In women, the WHR was positively associated with systolic blood pressure, diastolic blood pressure, and serum concentrations of serum total cholesterol. The prevalence of hypertension and hypertriglyceridemia increased as the WHR increased. The same results were not observed among men. The optimal WHR cutoffs for predicting hypertension, diabetes, dyslipidemia and ≥ two of these risk factors for Han adults in Xinjiang were 0.92, 0.92, 0.91, 0.92 in men and 0.88, 0.89, 0.88, 0.89 in women, respectively.

**Conclusions:**

Higher cutoffs for the WHR are required in the identification of Han adults aged ≥ 35 years with a high risk of cardiovascular diseases in Xinjiang.

## Background

Obesity is becoming an epidemic health problem worldwide in developed and developing countries [1]. The prevalence of obesity in adults increased by nearly 50% from the 1980s to 1990s [2]. Currently, approximately 70% of adults are classified as overweight or obese compared with a prevalence of 25% for obesity 40 years ago [3,4]. Overweight and obesity represent a rapidly growing threat to the health of populations in an increasing number of countries [1].

Obesity has also been shown to be associated with numerous cardiovascular disease (CVD) risk factors, such as hypertension, dyslipidemia, type 2 diabetes, and insulin resistance [5-8]. A simple anthropometric measurement as an indicator for obesity related to cardiovascular risk factors is of interest for use in public health actions. Many studies have reported that body fat distribution is a more powerful predictor than body mass index (BMI) for risk factors, diseases, and mortality [9,10]. In particular, increased visceral or abdominal adipose tissue is more strongly associated with metabolic and CVD risk and a variety of chronic diseases [11,12]. BMI is consistently associated with an increased risk of CVD and type 2 diabetes [13], but this measurement does not account for variation in body fat distribution and abdominal fat mass, which can greatly differ across populations and substantially vary within a narrow range of BMI [14]. Excess intra-abdominal fat is associated with a greater risk of obesity-related morbidity than overall adiposity [15,16]. Several studies in adults have reported a stronger positive association between cardiovascular risk factors and abdominal adiposity than overall adiposity [17,18]. Therefore, central obesity indices are slightly better than BMI regarding an association with cardiovascular risk factors [19,20].

The waist-to-hip ratio (WHR), a simple indicator for central obesity, has also been reported as a good surrogate for central obesity and is associated with the risk of CVD [20-25]. Similar to BMI, the relationship of WHR and related health risks is affected by age, sex, and regional differences. Therefore, the optimal cutoff values for WHR in detection of cardiovascular risks are likely to be different among different populations [26]. In 2002, a study in 11 provinces of China suggested that the optimal cutoffs of WHR in men and women were 0.90 and 0.80, respectively [27]. A study in Thailand suggested that the optimal cutoffs of WHR for diabetes, hypertension, and dyslipidemia were 0.89–0.91 in men and 0.85–0.88 in women [20]. For Chinese in Hong Kong, the optimal cutoff of WHR is 0.85 in men and 0.80 in women [22]. A previous study assessed optimal cutoffs for WHR in terms of association with diabetes, hypertension, and dyslipidemia in Taiwan [23]. The optimal cutoffs were 0.87–0.90 and 0.78–0.83 for WHR in men and women, respectively. In Jordanian adults, the WHR cutoff values vary from 0.88 to 0.90 in men and from 80.0 to 0.83 in women [24]. Therefore, applying the regional optimal cutoff of WHR for screening populations with a high risk of CVD is important.

Xinjiang Uighur Autonomous Region, which is located in the center of Asia and northwest of China, has a unique cultural and geographical background. The total Han population was 7.5 million in 2000, and 40.61% of the total population is in Xinjiang. The optimal cutoff of WHR associated with cardiovascular risk factors among Han adults in Xinjiang remains unknown. Therefore, in the present study, we examined the relationship between WHR and cardiovascular risk factors. We also determined the optimal cutoff points of WHR for detecting cardiovascular risk factors among Han adults in Xinjiang.

## Methods

### Ethics statement

The present study was approved by the Ethics Committee of the First Affiliated Hospital of Xinjiang Medical University and was conducted according to the standards of the Declaration of Helsinki. Written, informed consent was obtained from the participants.

### Sample design

All of the participants were selected from the Cardiovascular Risk Survey (CRS) study. The details of the study population and methods have been described previously [28,29]. Briefly, the CRS study used a four-stage stratified sampling method to select a representative sample of the general population in Xinjiang, northwest of China. The research sites included Urumqi City, Kelamayi City, Fukang City, Turpan Prefecture, Hetian Prefecture, and Yili Prefecture. The time period of the study was from October 2007 to March 2010. The selections made from sampling units were based on geographic area, sex, and age groups using household registries. The four-stage stratified sampling method was as follows. In stage one, according to population census data of Xinjiang in 2000, the areas mentioned above were selected based on population, ethnicity, geography, economic and cultural development level. In stage two, according to the ethnic aggregation status, one district or county was randomly selected from an area with a dominant Uighur population. In stage three, one community or town (village) was randomly selected from each district or county. In stage four, subjects aged older than 35 years were randomly selected from each community or town (village) as research subjects. Staff conducted surveys in households and administered questionnaires. The questionnaires included demographic, socioeconomic, and dietary information, and the medical history of each participant. In total, the CRS included 14,618 participants (5757 Hans, 4767 Uighurs, and 4094 Kazakhs).

A total of 5595 Han subjects with complete anthropometric data were enrolled in the present study. A total of 2700 subjects were men and 2895 subjects were women. The age of the subjects ranged from 35 to 88 years old, with a mean ± SD age of 52.52 ± 12.70 years (men, 52.31 ± 13.29 years; women, 52.72 ± 12.12 years).

### Anthropometric measurements

Data collection involved one visit in the participants’ residential areas. During the examinations, a standard questionnaire assessing demographic information and medical history was collected by trained research staff. A waist circumference measurement was taken at the end of normal expiration and to the nearest 0.1 cm, measuring from the narrowest point between the lower borders of the rib cage and the iliac crest. Hip circumference was measured to the nearest 0.1 cm, as recommended by the World Health Organization (WHO) [30]. The WHR was calculated.

### Laboratory methods

Blood samples were obtained from an antecubital vein into Vacutainer tubes containing EDTA in the morning after an overnight fasting period. Blood samples were centrifuged within two hours at the survey site. Plasma was transferred to separate labeled tubes and transported immediately on dry ice at prearranged intervals to the Xinjiang coronary artery disease VIP laboratory. Serum concentrations of serum total cholesterol, triglycerides, low-density lipoprotein (LDL), high-density lipoprotein (HDL), and fasting glucose were measured by the Clinical Laboratory Department of the First Affiliated Hospital of Xinjiang Medical University with a biochemical analyzer (Dimension AR/AVL Clinical Chemistry System, Newark, NJ, USA) [28,29].

### Blood pressure measurement

A mercury sphygmomanometer was used to measure blood pressure in the sitting position after a 10-minute rest period. During the 30 minutes preceding measurement, the subjects were required to refrain from smoking or consuming caffeine. The appearance of the first sound was used to define systolic blood pressure, and the disappearance of sound was used to define diastolic blood pressure [31]. Two readings each of systolic and diastolic blood pressures were recorded, and the average of each measurement was used for data analysis. If the first two measurements differed by more than 5 mmHg, additional readings were taken.

### Definition of risk factors

Hypertension was defined as self-reported use of antihypertensive medication within the past 2 weeks or an average systolic blood pressure ≥140 mm Hg, an average diastolic blood pressure ≥90 mm Hg, or both.

Diabetes was defined as fasting plasma glucose ≥7.0mmol/L, use of insulin or oral hypoglycemic agents, or a self-reported history of diabetes.

Total cholesterol concentrations >6.22 mmol/L (240 mg/dl) were defined as hypercholesterolemia. Triglyceride concentrations >2.26 mmol/L (200 mg/dl) were defined as hypertriglyceridemia. LDL cholesterol concentrations >4.14mmol/L (160 mg/dl) were defined as high LDL cholesterol. HDL cholesterol concentrations <1.04 mmol/L (40 mg/dl) were defined as low HDL cholesterol [27]. Dyslipidemia was defined as any one of the four lipids abnormalities mentioned above or self-reported use of antihyperlipidemic medication.

### Statistical analysis

Statistical analysis was conducted using SPSS version 16.0 for Windows (SPSS Inc., Chicago, IL, USA). Continuous variables are expressed as sex-specific means and standard deviations, and discrete variables are expressed as sex-specific proportions. Analysis of variance was used for continuous variables and the chi-square test was used for categorical variables. A value of *P* < 0.05 indicates a statistically significant difference. Age standardization was performed by the direct method by using the Han population according to the population census data of Xinjiang in 2000 [32] as the standard population. The sensitivity and specificity of each WHR level for the detection of hypertension, dyslipidemia, diabetes, and two or more of these risk factors were calculated by creating dichotomous variables for each WHR value. Additionally, the distance on the receiver operating characteristic (ROC) curve of each WHR value was calculated as the square root of [(1 - sensitivity)^2^ + (1 - specificity)^2^]. The WHR value with the shortest distance on the ROC curve was considered in the determination of optimal cutoffs. The overall performance of the WHR test for detecting cardiovascular risk factors was assessed by computing the area under the curve (AUC). An AUC of 1 is considered to have perfect discriminatory power, and an AUC of 0.5 suggests that the discriminatory power is no better than chance.

## Results

Baseline characteristics of the distribution of age categories and sample size of cardiovascular risk factors by WHR category between sexes are shown in Tables 1 and 2.

**Table 1:** Distribution characteristics of age categories among subjects by sex

**Sex**	**35-39 years**	**40-44 years**	**45-49 years**	**50-54 years**	**55-59 years**	**60-64 years**	**65-69 years**	**70-74 years**	**75-79 years**	**80-84 years**	**85-89 years**	**Total**
Men	363	586	423	244	253	200	201	272	101	43	14	2700
Women	332	594	369	329	314	316	328	212	71	27	3	2895

**Table 2:** Sample size of cardiovascular risk factors in men and women by WHR category

	**WHR < 0.75**	**0.75 ≤ WHR < 0.80**	**0.80 ≤ WHR < 0.85**	**0.85 ≤ WHR < 0.90**	**0.90 ≤ WHR < 0.95**	**0.95 ≤ WHR < 1.00**	**WHR ≥ 1.00**	**Total**
Men								
Blood pressure(n)	27	74	225	656	839	530	304	2655
Fasting glucose(n)	25	73	226	648	834	513	301	2620
Total cholesterol(n)	25	73	226	648	834	513	300	2619
LDL cholesterol(n)	27	74	226	645	815	510	296	2593
HDL cholesterol(n)	27	74	226	645	815	510	297	2594
Triglycerides(n)	25	73	226	648	834	513	300	2619
Women								
Blood pressure(n)	122	319	602	649	453	311	399	2855
Fasting glucose(n)	117	310	590	639	452	311	397	2816
Total cholesterol(n)	117	310	590	639	452	311	398	2817
LDL cholesterol(n)	120	315	587	643	446	307	381	2799
HDL cholesterol(n)	120	315	589	643	446	307	383	2803
Triglycerides(n)	117	310	590	639	452	311	398	2817

Compared with men, WHR levels were higher, with higher systolic blood pressure, diastolic blood pressure and serum total cholesterol concentrations in women. We did not notice any trends in men (Tables 3 and 4).

**Table 3:** Age-standardized CVD risk factors in men by WHR category

	**WHR < 0.75**	**0.75 ≤ WHR < 0.80**	**0.80 ≤ WHR < 0.85**	**0.85 ≤ WHR < 0.90**	**0.90 ≤ WHR < 0.95**	**0.95 ≤ WHR < 1.00**	**WHR ≥ 1.00**	***P*** **values**
Population distribution n(%)	28(1.04%)	75(2.78%)	232(8.59%)	670(24.81%)	855(31.67%)	531(19.67%)	309(11.44%)	
Systolic blood pressure(mmHg)	134.70 ± 19.19	128.97 ± 15.08	128.44 ± 18.61	131.57 ± 17.34	134.45 ± 18.02	137.66 ± 18.48	141.28 ± 21.35	<0.001
Diastolic blood pressure(mmHg)	86.19 ± 12.27	83.12 ± 15.31	82.85 ± 15.02	85.86 ± 14.42	87.42 ± 14.27	89.70 ± 15.90	91.06 ± 16.44	<0.001
Total cholesterol(mmol/L)	4.38 ± 0.68	4.53 ± 1.08	4.37 ± 1.01	4.63 ± 1.09	4.77 ± 1.10	4.72 ± 1.07	4.68 ± 0.92	<0.001
HDL cholesterol(mmol/L)	1.17 ± 0.42	1.30 ± 0.44	1.26 ± 0.39	1.24 ± 0.45	1.24 ± 0.47	1.21 ± 0.42	1.26 ± 0.43	0.473
LDL cholesterol(mmol/L)	2.59 ± 0.81	2.74 ± 0.68	2.80 ± 0.87	2.83 ± 0.88	2.88 ± 0.91	2.89 ± 0.92	2.88 ± 0.91	0.326
Triglycerides(mmol/L)	1.51 ± 1.02	1.36 ± 0.96	1.40 ± 1.14	1.78 ± 1.59	2.16 ± 1.78	2.12 ± 1.75	2.10 ± 1.35	<0.001
Fasting glucose(mmol/L)	5.30 ± 1.12	5.12 ± 1.52	5.25 ± 2.39	5.45 ± 1.69	5.40 ± 1.63	5.57 ± 2.41	5.80 ± 2.21	0.011

Data are presented as mean ± SD or n(%).

**Table 4:** Age-standardized CVD risk factors in women by WHR category

	**WHR < 0.75**	**0.75 ≤ WHR < 0.80**	**0.80 ≤ WHR < 0.85**	**0.85 ≤ WHR < 0.90**	**0.90 ≤ WHR < 0.95**	**0.95 ≤ WHR < 1.00**	**WHR ≥ 1.00**	***P*** **values**
Population distribution n(%)	124(4.28%)	325(11.23%)	608(21.00%)	658(22.73%)	462(15.96%)	315(10.88%)	403(13.92%)	
Systolic blood pressure(mmHg)	119.48 ± 18.42	119.76 ± 17.59	125.23 ± 18.45	131.4 ± 21.50	134.6 ± 19.59	136.41 ± 20.24	143.39 ± 19.22	<0.001
Diastolic blood pressure(mmHg)	78.27 ± 15.33	78.89 ± 15.13	80.81 ± 14.57	83.08 ± 16.00	84.22 ± 15.69	84.90 ± 16.40	86.17 ± 15.40	<0.001
Total cholesterol(mmol/L)	4.28 ± 1.30	4.48 ± 0.94	4.61 ± 1.04	4.70 ± 1.18	4.74 ± 1.05	4.85 ± 1.00	4.94 ± 1.10	<0.001
HDL cholesterol(mmol/L)	1.28 ± 0.43	1.28 ± 0.43	1.30 ± 0.50	1.26 ± 0.46	1.25 ± 0.40	1.26 ± 0.44	1.25 ± 0.56	0.54
LDL cholesterol(mmol/L)	2.83 ± 1.03	2.86 ± 0.93	2.87 ± 0.96	2.88 ± 0.92	2.91 ± 0.91	2.88 ± 0.95	2.90 ± 0.87	0.976
Triglycerides(mmol/L)	1.16 ± 1.67	1.16 ± 0.78	1.30 ± 0.87	1.48 ± 1.08	1.64 ± 1.42	1.61 ± 1.16	1.83 ± 1.16	<0.001
Fasting glucose(mmol/L)	4.83 ± 1.47	5.03 ± 1.33	5.07 ± 1.62	5.19 ± 1.39	5.32 ± 1.54	5.16 ± 1.62	5.62 ± 1.77	<0.001

Data are presented as mean ± SD or n(%).

In women, the prevalence of hypertension and hypertriglyceridemia increased as the WHR increased. The prevalence of diabetes, hypercholesterolemia, high LDL cholesterol, and low HDL cholesterol did not show any significant relationship with WHR. We did not observe any relationships with these variables and WHR in men (Tables 5 and 6).

**Table 5:** Age-standardized prevalence of risk factors in men by WHR category

	**WHR < 0.75**	**0.75 ≤ WHR < 0.80**	**0.80 ≤ WHR < 0.85**	**0.85 ≤ WHR < 0.90**	**0.90 ≤ WHR < 0.95**	**0.95 ≤ WHR < 1.00**	**WHR ≥ 1.00**	***P*** **values**
Hypertension	29.6%(14)	29.7%(25)	27.6%(67)	36.3%(243)	39.2%(334)	48.7%(259)	55.6%(174)	<0.001
Diabetes	8%(6)	5.5%(8)	5.8%(17)	9%(63)	9.1%(81)	10.1%(57)	12.3%(49)	0.205
Hypercholesterolemia	16%(9)	26%(20)	20.8%(52)	25.9%(177)	32.1%(274)	29.6%(159)	27.7%(97)	0.011
High LDL cholesterol	22.2%(11)	32.4%(26)	36.3%(88)	36.9%(242)	38.7%(318)	38.2%(203)	38.9%(119)	0.605
Low HDL cholesterol	40.7%(16)	28.4%(23)	28.3%(72)	34.7%(229)	35.3%(291)	37.6%(211)	31.6%(103)	0.169
Hypertriglyceridemia	32%(13)	23.3%(19)	22.6%(54)	35.2%(232)	49%(504)	52%(270)	52.7%(177)	<0.001

Data are presented as %(n).

**Table 6:** Age-standardized prevalence of risk factors in women by WHR category

	**WHR < 0.75**	**0.75 ≤ WHR < 0.80**	**0.80 ≤ WHR < 0.85**	**0.85 ≤ WHR < 0.90**	**0.90 ≤ WHR < 0.95**	**0.95 ≤ WHR < 1.00**	**WHR ≥ 1.00**	***P*** **value**
Hypertension	18.9%(29)	20.1%(73)	27.7%(162)	35.6%(241)	41.9%(197)	47.3%(149)	58.4%(230)	<0.001
Diabetes	3.4%(7)	4.2%(13)	3.4%(27)	6.7%(45)	9.1%(40)	6.1%(18)	13.4%(57)	<0.001
Hypercholesterolemia	20.5%(31)	20%(68)	26.3%(153)	30.2%(197)	30.8%(143)	37.3%(113)	37.7%(168)	<0.001
High LDL cholesterol	33.3%(48)	34%(110)	35.8%(214)	36.7%(242)	37.9%(171)	35.8%(115)	38.6%(159)	0 .849
Low HDL cholesterol	31.7%(51)	30.2%(90)	29%(178)	31.7%(203)	30.9%(144)	32.6%(98)	34.5%(144)	0 .717
Hypertriglyceridemia	12%(22)	15.2%(54)	18.5%(106)	26.8%(175)	31.6%(146)	32.2%(94)	44.5%(182)	<0.001

Data are presented as %(n).

The population distribution of each WHR level, and the sensitivity, specificity, and distance on the ROC curve for the detection of hypertension, dyslipidemia, diabetes, and ≥ two of these risk factors for men and women are shown in Tables 7 and 8, respectively. In men, the cutoff for dyslipidemia was 0.91. The shortest distances on the ROC curve of hypertension, diabetes and ≥ two of these risk factors were the same. A WHR of 0.92 appeared to be the optimal WHR cutoff in men. In women, the shortest distance on the ROC curve for hypertension and dyslipidemia was 0.88. The shortest distance on the ROC curve for diabetes and ≥ two of these risk factors was 0.89.

**Table 7:** Sensitivity(sens), specificity(spec), and distance in the ROC curve for WHR cutoffs in men

		**Hypertension**			**Diabetes**			**Dyslipidemia**			**≥2 risk factors**		
**WHR**	**n**	**Sens %**	**Spec %**	**Distance in ROC curve**	**Sens %**	**Spec %**	**Distance in ROC curve**	**Sens %**	**Spec %**	**Distance in ROC curve**	**Sens %**	**Spec %**	**Distance in ROC curve**
0.80	21	97.00	4.90	0.95	97.50	4.20	0.96	96.90	5.30	0.95	98.50	5.10	0.95
0.81	29	96.40	6.10	0.94	96.70	5.20	0.95	96.00	6.60	0.93	98.30	6.30	0.94
0.82	33	95.60	7.50	0.93	94.60	6.30	0.94	95.20	8.30	0.92	97.20	7.40	0.93
0.83	49	94.00	9.50	0.91	94.20	8.30	0.92	93.70	10.80	0.89	96.10	9.60	0.90
0.84	48	93.10	11.80	0.88	93.40	10.20	0.90	92.60	13.80	0.87	95.30	11.80	0.88
0.85	71	91.30	15.00	0.85	92.10	13.00	0.87	90.60	17.40	0.83	94.30	15.20	0.85
0.86	84	88.60	18.40	0.82	89.70	16.10	0.85	88.00	20.90	0.80	92.40	18.70	0.82
0.87	138	84.80	24.20	0.77	86.80	21.10	0.80	83.30	26.00	0.76	88.90	24.10	0.77
0.88	137	81.00	30.30	0.72	82.20	26.50	0.76	78.10	31.60	0.72	84.80	29.70	0.72
0.89	148	76.30	36.20	0.68	75.60	31.70	0.73	73.20	37.50	0.68	79.80	35.30	0.68
0.90	179	64.80	42.20	0.68	68.20	38.50	0.69	66.00	44.10	0.65	71.40	41.50	0.65
0.91	162	63.00	48.30	0.64	63.60	44.40	0.66	59.90	49.40	0.65	66.30	47.60	0.62
0.92	151	57.00	54.10	0.63	59.10	50.30	0.64	53.30	53.80	0.66	60.50	53.40	0.61
0.93	164	51.40	60.40	0.63	51.20	56.30	0.66	46.60	58.90	0.67	53.10	59.10	0.62
0.94	187	44.30	67.50	0.64	45.00	63.40	0.66	39.50	65.40	0.70	47.00	66.70	0.63
0.95	144	38.30	75.00	0.67	36.80	70.30	0.70	32.50	72.60	0.73	39.90	73.60	0.66

**Table 8:** Sensitivity(sens), specificity(spec), and distance in the ROC curve for WHR cutoffs in women

		**Hypertension**			**Diabetes**			**Dyslipidemia**			**≥2 risk factors**		
**WHR**	**n**	**Sens %**	**Spec %**	**Distance in ROC curve**	**Sens %**	**Spec %**	**Distance in ROC curve**	**Sens %**	**Spec %**	**Distance in ROC curve**	**Sens %**	**Spec %**	**Distance in ROC curve**
0.80	92	91.30	20.70	0.80	91.20	16.50	0.84	85.90	17.90	0.83	93.70	19.00	0.81
0.81	76	88.60	23.30	0.78	90.20	19.30	0.81	83.70	21.10	0.81	91.20	21.80	0.79
0.82	122	85.80	28.50	0.73	88.60	23.60	0.77	79.90	25.70	0.77	89.40	26.70	0.74
0.83	139	82.40	34.10	0.68	86.50	28.60	0.73	76.00	31.30	0.73	86.70	32.10	0.69
0.84	142	78.80	39.80	0.64	82.40	33.50	0.69	71.80	37.00	0.69	83.60	37.60	0.65
0.85	117	75.50	44.20	0.61	80.80	37.80	0.65	68.00	41.30	0.67	80.30	41.80	0.61
0.86	114	72.20	48.60	0.58	78.80	41.90	0.62	64.20	45.30	0.65	77.20	46.00	0.59
0.87	138	67.10	53.20	0.57	73.10	46.60	0.60	59.90	50.50	0.64	72.40	50.90	0.56
0.88	151	62.20	58.60	0.56	67.90	51.70	0.58	54.70	55.80	0.63	67.70	56.10	0.55
0.89	126	57.70	62.40	0.57	64.20	55.90	0.57	50.00	59.40	0.64	63.00	60.10	0.54
0.90	127	53.70	67.40	0.57	57.00	60.30	0.59	45.30	64.00	0.65	58.00	64.50	0.55
0.91	94	49.30	69.90	0.59	51.30	63.40	0.61	41.60	67.00	0.67	54.20	67.70	0.56
0.92	110	45.60	73.80	0.60	47.70	67.20	0.62	37.20	70.10	0.70	50.50	71.50	0.57
0.93	80	42.40	76.30	0.62	42.50	69.80	0.65	34.50	73.20	0.71	46.80	74.00	0.59
0.94	84	39.10	78.90	0.64	40.40	72.80	0.66	31.50	76.10	0.73	44.40	77.10	0.60
0.95	94	35.90	81.80	0.67	37.30	75.90	0.67	28.10	78.60	0.75	41.00	79.90	0.62

**Figure 1:**
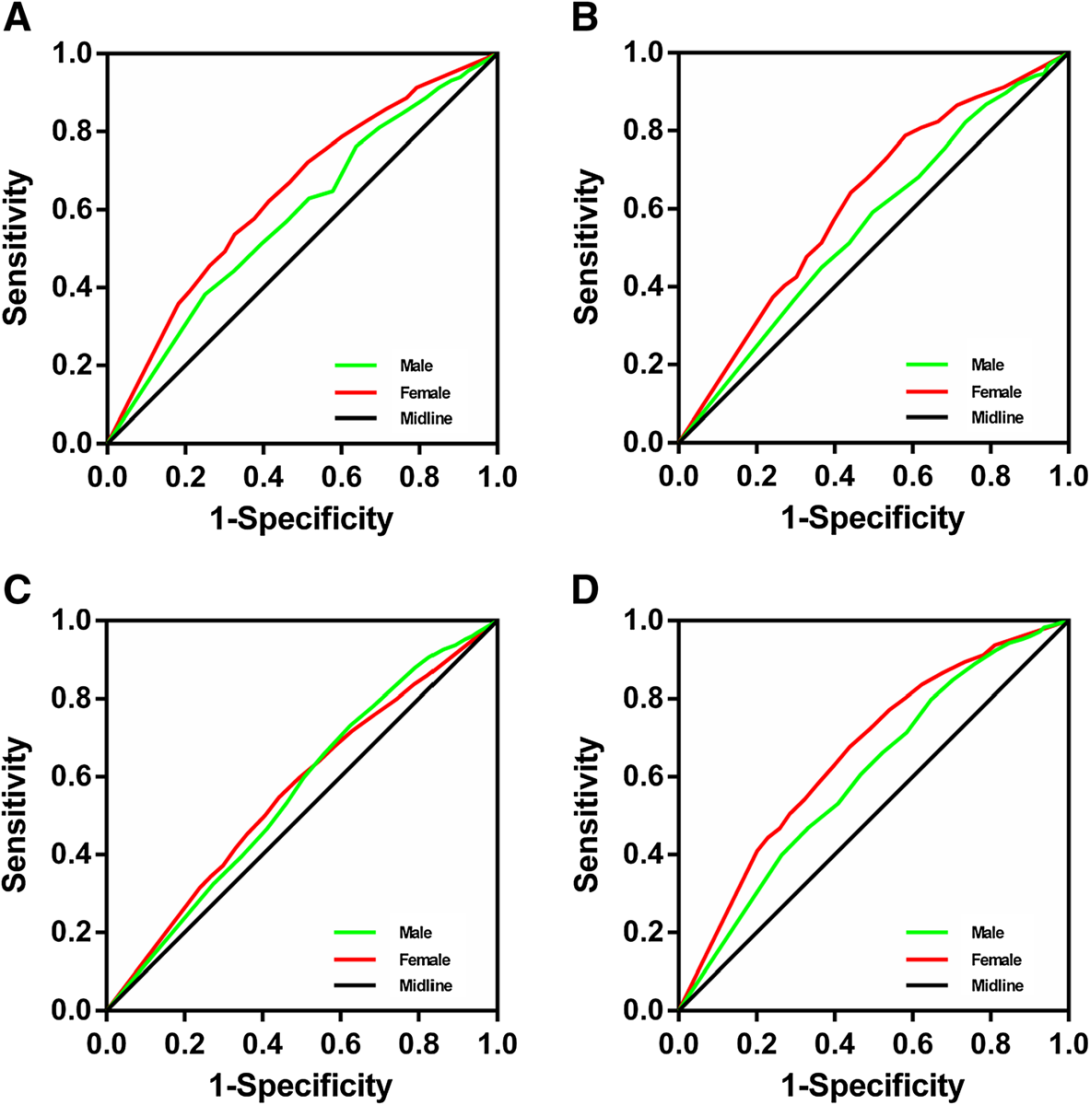
**ROC curves to detect CVD risk factors by sex. (A)** ROC curves for both men and women for the detection of hypertension, **(B)** diabetes, **(C)** dyslipidemia and **(D)** ≥ 2 of these risk factors.

The ROC curves for men and women are shown in Figure 1. The AUCs of each cardiovascular risk factor in men and women are shown in Table 9. We found that the discriminatory power of WHR for cardiovascular risk factors was slightly better in women than in men.

**Table 9:** AUC of each cardiovascular risk factor in men and women

	**AUC(95% CI) in men**	**AUC(95% CI) in women**
Hypertension	0.590(0.568, 0.612)	0.645(0.624, 0.665)
Diabetes	0.561(0.524, 0.599)	0.629(0.588, 0.669)
Dyslipidemia	0.553(0.530, 0.576)	0.562(0.540, 0.583)
≥2 risk factors	0.606(0.583, 0.630)	0.665(0.642, 0.688)

## Discussion

Obesity is becoming an epidemic health problem worldwide in developed and developing countries [1]. In 1998, the WHO announced that the cutoffs of WHR used to define central adiposity were 0.90 in men and 0.85 in women [14]. According to the WHO cutoffs for the designation of central adiposity based on Western populations, the present study showed that 62.8% of male Han adults had a WHR ≥0.90 and 63.5% of the female Han adults had a WHR ≥0.85.

Obesity, especially abdominal adiposity, has been shown to increase the risk of CVD through many cardiovascular risk factors, including hypertension, diabetes, and dyslipidemia [5-8]. WHR, a simple indicator for central obesity, has also been reported as a discriminator for identifying individuals with CVD risk factors [20,25]. Despite the fact that a close relationship is apparent between abdominal adiposity and the risk of CVD, the current WHR cutoffs suggested by the WHO are not based on associations with CVD risk factors, but rather on their correlation with corresponding values of BMI. In the Asian population, which has a predisposition to central obesity and related increased risk in CVD, the WHR had been set with different cutoff points across regions [20,22-24]. Therefore, determining the optimal cutoff of WHR based on associations with CVD risk factors among Han adults in Xinjiang to allow effective screening is important.

In the present study, based on the sensitivity, specificity, and ROC calculations, the optimal cutoffs of WHR for Han men and women in Xinjiang were 0.92 and 0.89, respectively. We found that although the Han population in Xinjiang belonged to the Asian population, the optimal cutoffs of WHR for CVD risk factors among Han adults in Xinjiang were higher than those in some other regions of Asia, such as Hong Kong [22], Taiwan [23], Thailand [20], and Jordan [24]. The reasons why there are higher optimal cutoffs of WHR among Han adults in Xinjiang are unclear. Possible reasons may be associated with differences in diet, climate, and some important genes that regulate body fat distribution. First, the primary reason may be a difference in diet in Han adults compared with Asian populations in other regions. The Han population in Xinjiang consumes more pasta, meat, and milk products than Asian populations in other regions. Second, a characteristic feature of the climate in Xinjiang is that the temperature difference during day and night is considerable, and winter is extremely cold. Therefore, subcutaneous fat thickness in the Han population in Xinjiang may be thicker than other populations for adaptation to the external environment. Third, certain genes that regulate body fat distribution may be different in different populations, so that body fat is more likely to accumulate in the abdomen [33-35]. In addition, the difference in optimal WHR cutoffs between populations might be due to differences in body size, metabolic status, and physical activity. We also found that the optimal cutoffs of WHR for detecting cardiovascular risk factors were higher than WHO cutoffs for the definition of central adiposity. Apart from ethnic and regional differences, the main reason for this difference may be that the definition of central adiposity was not based on associations with CVD risk factors. Moreover, the WHO definition was published nearly a decade ago, and the prevalence of obesity among adults increased by nearly 50% during the 1980s and 1990s, and the current prevalence of obesity is nearly three higher compared with 40 years ago. Therefore, as the prevalence of obesity worldwide increases [36,37], WHR cutoffs may have some minor changes compared with definitions made 15 years ago.

In addition, we also found that in men with a WHR <0.75, the prevalence of low HDL cholesterol was up to 40%, which was significantly higher than that in men with other WHR levels. The prevalence of diabetes and hypertriglyceridemia also appeared to be higher in men with a WHR <0.75. These findings could be attributed to the following factors. First, the number of subjects in men with a WHR <0.75 (only 28 subjects) was less than that in other groups. Therefore, the power of the data set may be low. Second, most of subjects in men with a WHR <0.75 were likely to suffer from type 1 diabetes, glucose metabolism disorders, and other diseases, which may result in dyslipidemia. Third, the majority of subjects in men with a WHR <0.75 may have a history of long-term heavy smoking, excessive drinking, and malnutrition.

The present study has several strengths. This is the first representative sample of general Han adults in Xinjiang. Therefore, these results can be generalized to adults of the Han population aged older than 35 years. Additionally, we provided information for a wide range of WHR values, stratified by sex. Future studies can use these WHR cutoffs to further study the associated risk factors and intervention of overweight and obesity in a representative sample of the Han adult population in Xinjiang. There are also limitations of the present study. First, our study was a cross-sectional design. Therefore, cause-effect temporality could not be readily evaluated. Second, because of a lack of information, we could not exclude potential confounding effects from medications, such as glucocorticoids, or from the presence of liver cirrhosis, thyroid disease, ascites, malnutrition, and cancer. Third, in data analysis, we excluded outliers and missing values in blood specimens caused by systemic factors, which can lead to incomplete data of blood samples, and affect the results.

## Conclusions

This study showed WHR values of 0.92 in men and 0.89 in women as the optimal cutoffs in the identification of Han adults at a high risk of CVD. This study has added further important knowledge on the relationship between WHR values and cardiovascular risk factors.

## Competing interests

The authors declare that they have no competing interests.

## Authors’ contributions

SSL, SP, YTM, and YNY conceived and designed the experiments. SSL, SP, YTM, YNY, XM, XML, ZYF, XX, FL, YC, BDC, ZXY, CHH, YYZ, NA, JA, and YTW performed the experiments. SSL, SP, and FL analyzed the data. YTM, YNY, XM, XML, ZYF, and XX contributed reagents, materials, and analysis tools. SSL, SP, and YTM wrote the manuscript. All authors read and approved the final manuscript.

## Pre-publication history

The pre-publication history for this paper can be accessed here:

http://www.biomedcentral.com/1471-2261/14/93/prepub
